# Increased volume and impaired function: the role of the basal ganglia in writer’s cramp

**DOI:** 10.1002/brb3.301

**Published:** 2014-12-24

**Authors:** Kirsten E Zeuner, Arne Knutzen, Oliver Granert, Julia Götz, Stephan Wolff, Olav Jansen, Dirk Dressler, Harald Hefter, Mark Hallett, Günther Deuschl, Thilo van Eimeren, Karsten Witt

**Affiliations:** 1 Department of Neurology, Kiel UniversityKiel, Germany; 2 Department of Neuroradiology, Kiel UniversityKiel, Germany; 3 Movement Disorders Section, Department of Neurology, Hannover Medical SchoolHannover, Germany; 4 Department of Neurology, University of DüsseldorfDüsseldorf, Germany; 5 Human Motor Control Section, Medical Neurology Branch, National Institute of Neurological Disorders and Stroke, National Institutes of HealthBethesda, Maryland

**Keywords:** Focal hand dystonia, functional magnetic resonance imaging, motor learning, voxel-based morphometry, writer's cramp

## Abstract

**Introduction:**

The pathophysiology of writer's cramp, a task-specific dystonia, remains unclear. The objective of this study was to investigate the basal ganglia circuit and the cerebellum during a complex motor sequence learning task carried out with the nonaffected hand in writer's cramp patients.

**Methods:**

We applied structural and functional imaging in 22 writer's cramp patients and 28 matched controls using 3T MRI. With the asymptomatic left hand all participants learned a complex, sequential, five-element sequence-tapping task as accurately and quickly as possible. Functional imaging was measured during a repeated (15 times), fixed block design with tapping (30 sec) and rest (30 sec). Additionally, gray matter volume of the basal ganglia was analyzed using voxel-based morphometry (VBM).

**Results:**

While behavior was comparable between groups, after small volume correction the anterior part of the right putamen and the left globus pallidus exhibited reduced blood oxygen level-dependent (BOLD) activity in patients during the sequential finger-tapping task. VBM analysis showed larger gray matter volume bilateral in the posterior part of the putamen and globus pallidus. There were no group differences in the cerebellum.

**Conclusion:**

The results indicate an impairment of anterior basal ganglia loops involved in producing complex sequential movements of the unaffected hand. These findings are in line with previous reports of reduced neuronal activity in the globus pallidus internus. Higher gray matter volume of the putamen and globus pallidus may stem from elevated activity of the direct pathway, which could reflect a compensatory phenomenon or a primary predisposition, that is, endophenotypic trait.

## Introduction

Task-specific dystonia is a movement disorder that is characterized by cocontraction of antagonist muscles while performing a specific task. The most common forms are writer's and musician's cramp (Defazio et al. 2007). The underlying pathophysiology of task-specific dystonias remains unclear, but seems to be associated with abnormal patterns of activity at multiple levels within the sensorimotor system (Berardelli et al. 1998; Hallett 1998, 2006; Meunier et al. 2003; Peller et al. 2006). The results of several functional and structural neuroimaging studies that investigated the pathophysiology of task-specific dystonia in the basal ganglia (Blood et al. 2004; Delmaire et al. 2005), and the sensorimotor cortex (Bara-Jimenez et al. 1998; Elbert et al. 1998; Meunier et al. 2001, 2003; Garraux et al. 2004), have been contradictory. This has been attributed to the differences in motor tasks, techniques, and patient selection that were implemented among the studies. Most functional magnetic resonance imaging (fMRI) studies used motor paradigms that may induce dystonia (e.g., scribbling with the affected hand in writer's cramp) and described increased or decreased activation of the sensorimotor cortex, supplementary/premotor areas, cerebellum, or thalamus (Odergren et al. 1998; Ibanez et al. 1999; Pujol et al. 2000; Preibisch et al. 2001; Lerner et al. 2004; Havrankova et al. 2012). Activations of these regions have been difficult to interpret as they could have resulted from impaired motor performance of the affected limb tested. A few studies employed simple finger tapping of the affected side to avoid dystonia-related variability (Ibanez et al. 1999; Oga et al. 2002; Islam et al. 2009; Wu et al. 2010). The most recent study in musician's cramp with a nondystonic finger-tapping task demonstrated less activation in patients in the posterior parietal cortex, anterior putamen and the cerebellum, the sensorimotor cortex, and supplementary motor area (Wu et al. 2010). Complexity and frequency of motor tasks also differed among the studies and might explain, in part, the divergent results, because they are differentially processed in the motor, premotor, and associative basal ganglia territories (Lehericy et al. 2005). In patients with dystonia, the corticostriatal pathway seems to be functionally impaired and this malfunction is compensated by the corticocerebellar system (Doyon and Benali 2005; Doyon 2008). In a positron emission tomography (PET), the concept has been confirmed in nonmanifesting DYT1 carriers, who showed overactivity in the lateral cerebellum and the right inferotemporal cortex to achieve successful sequence learning (Ghilardi et al. 2003; Carbon et al. 2008).

Besides using functional imaging techniques, studies examining the morphometric changes in focal dystonia also reported variable results. Either increased or decreased gray matter volume, in some studies bilaterally, have previously been described for the putamen or globus pallidus in patients with different types of focal dystonia (Black et al. 1998; Etgen et al. 2006; Egger et al. 2007; Bradley et al. 2009; Granert et al. 2011; Pantano et al. 2011).

Although clinical manifestations in focal hand dystonia are typically unilateral in the beginning, the disorder is considered as a bilateral dysfunction of the basal ganglia (Hallett 2011). This is demonstrated by the fact that up to 25% of patients develop bilateral focal hand dystonia, if they switch to the previously unaffected hand (Sheehy and Marsden 1982; Zeuner and Molloy 2008).

In contrast to previous studies, writer's cramp patients in this study were asked to perform a non-task-related paradigm with the nonaffected hand to ensure that dystonic symptoms do not interfere with the motor performance. A sequential finger-tapping task was chosen, because it is considered a well-known, established task, is non-task specific, and is regarded to be complex (Walker et al. 2003a; Verstynen et al. 2005; Wu et al. 2010). This should give us critical insight into the primary pathophysiology of the basal ganglia in this condition. Further differences to earlier imaging studies comprised a complex motor task that contained sequential finger tapping and not writing. Finally, we advanced recent studies by investigating the basal ganglia circuit in two ways, structurally and functionally by combining voxel-based morphometry (VBM) and fMRI. Based on the studies mentioned above, we hypothesized that the corticostriatal circuit showed reduced activation with emphasis in the putamen and globus pallidus and expected compensatory activity in the cerebellum. Parallel to the abnormal functional activities in the striatum, we further expected increased gray matter volume in the same areas, based on previous findings (Granert et al. 2011). Our main interest was to investigate this area in two different aspects by applying these two different methodologies.

## Methods

### Patients and controls

Twenty-five patients with writer's cramp (13 women) with a mean age of 50.9 ± SD 11.6 years (range: 27–70 years) and a mean disease duration of 14.2 ± SD 7.6 years (range: 3–36 years) volunteered for the study. Seven patients presented with simple writer's cramp with only writing being affected, while 15 patients exhibited dystonic cocontraction when performing fine motor tasks other than writing (complex writer's cramp). Sixteen patients had previously been treated with botulinum neurotoxin. In seven of those 16 patients, the last injection was applied more than 3 years before inclusion into the study. Thirty-one age-matched healthy individuals (14 women) with a mean age of 49.1 ± SD 8.0 (range: 30–68 years) served as controls. The diagnosis of writer's cramp was established by medical history and standard neurological examination including a writing test of the right and therefore affected hand. The last botulinum toxin injection was performed at least 3 months before inclusion. Exclusion criteria were any neurological or psychiatric disorder other than writer's cramp, a history of any impairment to the central nervous system, musicians, and professional typists. A tapping performance (tapping frequency and number of correct sequences) outside the 95% confidence interval for the mean was also an exclusion criterion. All participants gave written informed consent before the study. The study was conducted in full accordance with the Declaration of Helsinki and had been approved by the local ethics committee in Kiel.

### Clinical assessment of writer's cramp

Patients were videotaped while writing the German sentence “Die Wellen schlagen hoch” (“The waves are surging high”) 10 times without a break between consecutive sentences, and the severity of writer's cramp was analyzed from the video segments (face not shown) using the Writer's Cramp Rating Scale (WCRS; Wissel et al. 1996). A higher total WCRS score (with a maximum score of 30 points) implies more severe dystonic signs during handwriting.

The Arm Dystonia Disability Scale (ADDS) contains seven items that estimate the impairment of manual skills reported by patients. A score of 100% indicates normal motor function. The final score represents the percentage of normal manual activity. Therefore, a lower ADDS score denotes more severe functional impairment (Fahn 1989).

### Sequential finger-tapping task

**Figure 1:**
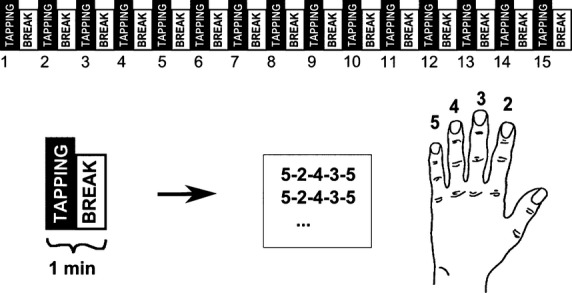
Timing of the functional magnetic resonance imaging (fMRI) experiment: The design included 15 blocks with a total duration of 15 min. Each block lasted 1 min, 30-sec active periods altered with 30-sec rest periods. Patients and controls pressed four keys on a manual hand device with the second to fifth fingers of their left (nondominant and not dystonic) hand, repeating the five-element sequence 5-2-4-3-5 as quickly and accurately as possible.

Patients and controls were required to press four keys on a manual hand device that was part of the Invivo IFIS fMRI system. They tapped with the second to fifth finger of their left (nondominant and not dystonic) hand, repeating the five-element sequence 5-2-4-3-5 for a period of 30 sec, as quickly and accurately as possible. The 30-sec trials alternated with 30-sec rest periods and lasted a total of 15 min, which corresponds to 15 repetitions (Fig.1). Before the participants were examined in the scanner, the motor task was demonstrated to them and they performed a few trials to assure that they understood the task. There was no formal training of the task prior to scanning. The numeric sequence 5-2-4-3-5 was displayed to the subjects through a mirror within the MRI scanner during the whole experiment to minimize working memory load. Each key press produced a switch from a fixation cross into a circle that lasted 100 msec on the screen. There was no accuracy feedback provided to the participants. The timing and precision of the key press responses were recorded for further analysis. The analysis included counting the total number of taps and the correctly completed sequences achieved for each of the 30-sec trials. The scores (speed and number of correct sequences) were averaged for each time point over all patients and separately over all control subjects (Walker et al. 2003a,b).

### MRI data acquisition

Anatomical and functional images were acquired in the Neurocenter at Kiel University hospital using a 3 Tesla whole-body MRI scanner (Achieva; Philips, Best, the Netherlands) equipped with an 8-channel head coil. An IFIS system (Invivo, Gainesville, FL) was used for stimulus presentation and response recording.

### Functional MRI

We performed a whole-brain echo planar imaging (EPI) to measure regional changes in the blood oxygen level-dependent (BOLD) signal. The EPI sequence consisted of 360 volumes with 38 axial slices. The slice thickness was 3.0 mm with an interslice gap of 0.3 mm. Axial slices were acquired parallel to the anterior–posterior plane. The other acquisition parameters of the functional MRI sequence were as follows: TR 2500 msec; TE 36.4 msec; FOV 216 × 216 × 125.1 mm^3^; matrix 64 × 64; flip angle 90 degree.

### Structural MRI

A three-dimensional (3D) T1-weighted gradient echo MRI scan with sagittal volume excitation was acquired from each participant with the following parameters: TR 7.8 msec; TE 3.6 msec; TI 800 msec; flip angle 8 degree; FOV 160 × 240 × 240 mm^3^; 160 slices with an image matrix and a scan resolution of 240 × 224 voxels and a reconstruction matrix of 256 × 256 voxels yielding in final voxel size of 1 × 0.94 × 0.94 mm^3^.

### Analysis of imaging data

Data preprocessing and statistical analysis was done with SPM8 (Release V4010) software (http://www.fil.ion.ucl.ac.uk/spm/) and Matlab 7.11.0 (MathWorks Inc., Natick, Massachusetts, USA).

### Image preprocessing

For spatial normalization of the images to the Montreal Neurological Institute (MNI) standard space the SPM8 segmentation algorithm was applied to the individual T1-weighted images. The functional EPI images of all three runs were registered to their mean using the SPM two pass realignment procedures. This realignment compensated for movements of the subjects during the scan sessions. We coregistered the mean image of the realigned EPI images to the corresponding individual T1-weighted image and used the concatenated transformation from this coregistration and the normalization from the T1 segmentation step to write normalized versions of the EPI images (re-sliced to a resolution with a voxel size of 2 × 2 × 2 mm^3^). This procedure optimized spatial normalization, because the complex nonlinear spatial normalization functions were determined from the high-resolution T1-weighted structural image and not from the EPI images with lower resolution and less contrast.

Finally, a smoothing filter with a Gaussian kernel of 8 mm full-width half-maximum (FWHM) was applied to the normalized EPI images to reduce residual anatomical differences and implement the Gaussian random field theory in further statistical analysis.

### Statistical analysis

#### Sequential finger-tapping task

Two separate two-factorial analysis of variance (ANOVAs) were computed to assess changes of the tapping frequency and the number of correct responses comprising the *number of blocks* as within-subject factor and *group* (patients vs. controls) as between-subjects factor. If necessary, Greenhouse-Geisser method was used to correct for nonsphericity. Conditional on the respective significant *F*-value, post-hoc paired (within-subject factor) *t*-tests were used to explore the direction of main effects. A *P*-value of 0.05 or less was considered significant. The sequential finger-tapping task measures fast motor learning (Walker et al. 2002, 2005). In addition to the ANOVA analysis, we performed a subanalysis and assessed motor learning performance (time course of correct sequences per block) in each individual to identify participants with learning deficits. We adopted a parametric formulation for a learning curve developed by Hull and Noble (Hull 1952; Noble 1957) to model trial-and-error learning. We adjusted their model and changed the formula as follows: NoCorrSeq_*n*_/*C* = *a* (*I*/*C*)^*r*^*n*^ which describes the learning motor performance (NoCorrSeq_n_) for each block (*n* = 1…15) by the asymptotic performance (*a*) and the exponential behavior (*r*) based on a constant of initial performance (*I*). We used the constant *I *=* *7.72 (corresponds approximately to 30% of the maximal performance) to incorporate the hypothetical initial performance, which we set as the average number of correct sequences in the first block for all participants. The second normalization constant *C* = 24 corresponded to the maximal number of correct sequences in a block tapped by any participant. We implemented a normalization to scale motor performance into an interval between 0 and 1. A nonlinear least square optimization procedure (function nls from the stats package in R statistics software) was chosen to determine the two individual parameters (*a* and *r*) for each participant. Based on this model, we identified subgroups with either “good” (*r* parameter < 0.99) or “insufficient” (*r* parameter ≥ 0.99) learning performance, which resulted in 18 sufficient learners in the patient group and 18 sufficient learners in the control group. While the parameter *r *=* *1 indicated constant performance, *r *<* *0.99 ensured that only subjects with a clear increase of the learning curve were defined as good learners.

#### Functional analysis (fMRI)

According to the experimental design described in the “Sequential Finger Tapping Task” section above, we used a basic first level fMRI block design model with 15 × 30 sec blocks of finger-tapping task that alternated with blocks of 30-sec rest. Additionally, we specified two further models each with one modulating regressor. The first model comprised for the tapping frequency, the second model of the number of correct sequences per block. These two additional models were used to assess the neuronal changes in relation to the training effect over time and had to be performed in two different models, because “frequency” is a parameter for motor speed, while “number of correct sequences” measures accuracy. Therefore, both parameters are closely correlated and estimates cannot be interpreted directly in a common statistical model. T-contrasts were specified to determine individual differences of the BOLD effect between the task and rest condition and to access the BOLD modulation with both, the tapping frequency and the number of correct sequences. Finally, we set up 3-sec level models to assess group differences between the patient and control group using the contrast images from the three-first level models. To compensate for possible age- or gender-related effects, we included an additional variable of no interest with subject's age and gender.

For the patient group, a second-level regression model including age and disease duration was evaluated to determine the relationship between disease duration and BOLD activity.

We performed an additional analysis to identify difference in BOLD signal at the beginning compared to the end of the learning process. For this purpose, the experiment of 15 blocks was divided into two parts, the first part consisting of blocks 1–7, and the second part from block 9 to 15. The BOLD effect in the tapping conditions of the second part was subtracted from the BOLD effect in the tapping conditions of the first part. We performed two different types of analysis to assess the learning effect: The first model directly compared the BOLD effect between the first and second part of the tapping experiment (Grafton et al. 2002; Floyer-Lea and Matthews 2005; Sun et al. 2007), while in the second model the block differences of the BOLD effect were related to the learning parameter *r* (as described in the sequential finger-tapping task section above) to analyze individual variations. In both cases, we looked at the overall effect and possible group differences. Additionally, we performed the above-mentioned group analyses with only those subjects that show sufficient learning performance. With this analysis, we ensured that subjects, who were not able to execute the task adequately, do not confound the results. In each group, we identified 18 sufficient learners by applying the above-mentioned criteria on the learning parameter (*r *<* *0.99).

#### Voxel-based morphometry (VBM)

The local gray matter maps from the segmentation step were modulated (multiplied by the local Jacobian of the deformation field) to get gray matter maps that represent local volume measurements. Resulting maps were smoothed with a 12 mm (FWHM) Gaussian kernel using the SPM8 software. The two groups were statistically compared by specifying a generalized linear model with a group factor (two levels: patients and controls) and three additional covariates. These covariates age, total brain volume, and gender (coded by the values −1 and 1) were added to compensate for gray matter variance due to the differences in head size and normal aging effects. Since we were also interested in the progression of the local gray matter volume during the course of the disease, we set up a regression model that included only the patient group and comprised the variable writer's cramp duration as well as the nuisance variables age, total brain volume and gender. Since age and disease duration showed no correlation, interfering effects could be excluded. Resulting statistical maps were analyzed with the same procedure and statistical thresholds as used in the fMRI analysis.

#### Threshold of significance for fMRI and VBM

We were particularly interested in the putamen and globus pallidus, because differences in BOLD activity and in gray matter volume have previously been described for these areas (also by our group) in patients with focal dystonia (Black et al. 1998; Blood et al. 2004; Delmaire et al. 2005; Etgen et al. 2006; Egger et al. 2007; Bradley et al. 2009; Granert et al. 2011; Pantano et al. 2011), and play an important part in the pathophysiology in this patient group (Hallett 2011). Therefore, we applied a family wise error correction for multiple comparisons (FWE, with *P* < 0.05) for our volume of interest by combining the bilateral volumes of the globus pallidus and putamen as defined by the AAL atlas (Tzourio-Mazoyer et al. 2002). We also applied FWE correction (*P* < 0.05) at the whole-brain level in order to detect any significant changes outside this primary volume of interest.

## Results

### Performance analysis of the sequential finger-tapping task

**Figure 2:**
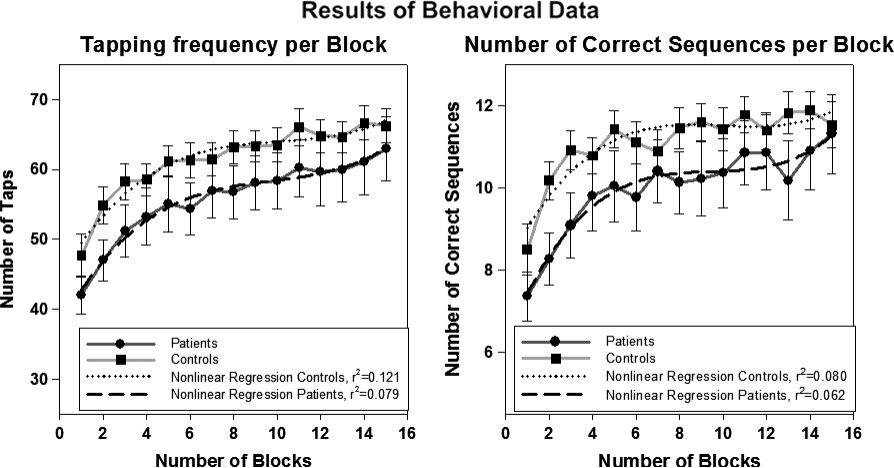
Behavioral data are shown for patients and controls (±SEM). The left panel shows the results for the tapping frequency per block, the right panel of the number of correct answers per block. The dark gray line presents writer's cramp patients (circles), the light gray line controls (squares). The nonlinear regression curves (cubic binominal curve fit) visualize the increase in tapping frequency (controls: *r*^2^* *=* *0.121; patients: *r*^2^* *=* *0.079) and the number of correct responses (controls: *r*^2^* *=* *0.080; patients: *r*^2^* *=* *0.062) during the time course of the experiment. Both groups improved significantly in tapping frequency and number of correct sequences over time (*P* < 0.0001), but there was no group difference (*P* = 0.154).

The behavioral data of five participants lay outside the 95% confidence margin (three controls and two patients) and these participants were excluded. One further patient had to be excluded due to multiple small vascular lesions that also affected the basal ganglia. Finally, 22 patients with writer's cramp (13 females) with a mean age of 50.7 ± SD 12.2 years (range: 27–70 years) and a mean disease duration of 14.2 ± SD 7.6 years and 28 age-matched healthy controls (11 females) with a mean age of 51.8 ± SD 7.4 (range: 33–68 years) were analyzed. Details of the patients’ characteristics are given in Table 1. All patients and controls were right handed (laterality quotient: patients 94.90 ± SD 6.95 [range: 77.8–100]; controls 89.63 ± SD 9.58 [range: 68.4–100.0]) according to the Oldfield handedness test (Oldfield 1971). The handedness test was not performed in patients P108 and P125. The ANOVA for repeated measurements that included the factor group (patients and controls) and the tapping frequency as a repeated measurement, revealed a main effect for *number of tapping blocks* (*F*_4.23_,_203.104_ = 40.0; *P* < 0.001; Fig.2), but not for group (*F*_1,48_ = 1.80; *P* = 0.19), and no interaction time × group (*P* = 0.76). The tapping frequency ascended in controls from 47.68 ± 16.13 to 66.21 ± 12.91 (mean ± SD), and in patients from 42.09 ± 12.78 to 63.00 ± 21.44 (mean ± SD). The mean number of correct sequences increased from 8.50 ± 3.26 to 11.54 ± 2.96 in controls and from 7.36 ± 2.88 to 11.32 ± 4.52 in patients. Both groups improved significantly over the blocks (*F*_5.14_,_246.67_ = 14.88; *P* < 0.001, Fig.2) and there was no group difference (*P* = 0.16). The analysis detected no interaction between the increase in number of correct sequences over time × group (*P* = 0.41). This means that motor learning was comparable in controls and patients. According to the work of Albouy et al. (2012) the interindividual variability was calculated and statistically compared between the groups. Using the two sample *t*-test, no differences between the two groups (*t*_24.2_ = −1.182, *P* = 0.25) were detected. The same procedure was executed for the asymptotic performance a (*t*_41.09_ = 1.158, *P* = 0.25) and the exponential behavior *r* (*t*_47.81_ = 0.4147, *P* = 0.68) and resulted in no group differences.

**Table 1:** Clinical data of patients with writer’s cramp included in the analysis

Patient ID	Age (years)	Symptom duration (years)	Type of writer's cramp	Last injection (months)	Duration BoNT treatment (years)	Total ADDS score (%)	Total WCRS score
P101	69	9	Complex	4	8	60.0	6
P103	52	7	Simple	14	0.25	72.9	10
P105	68	7	Simple	3	3.75	77.1	19
P106	49	10	Complex	n.a.	0	81.4	5
P107	43	9	Complex	60	3	68.6	22
P108	36	11	Simple	n.a.	0	64.3	8
P109	27	13	Complex	n.a.	0	55.7	10
P110	60	14	Simple	n.a.	0	72.4	6
P111	39	12	Simple	n.a.	0	51.4	17
P112	36	3	Complex	17	0.7	55.7	7
P115	56	14	Simple	5	10	60.0	10
P117	70	15	Complex	n.a.	0	68.6	13
P118	52	21	Complex	120	2	55.7	11
P119	35	21	Complex	36	0.5	48.9	14
P121	42	10	Complex	96	0.5	55.7	11
P122	59	13	Complex	120	0.5	51.4	6
P123	68	25	Complex	132	0.5	25.7	8
P124	50	17	Complex	96	3	42.9	8
P125	55	4	Complex	18	3	81.4	5
P127	51	19	Complex	3	4	72.9	3
P128	57	36	Complex	10	2	34.3	12
P129	42	22	Simple	6	1	68.6	11
Mean	50.7	14.1		46.2	2.6	58.7	10.1
SD	12.2	7.3		49.2	2.7	14.4	4.8

ADDS, Arm Dystonia Disability Scale; WCRS, Writer's Cramp Rating Scale; SD, standard deviation; BoNT, Botulinum neurotoxin.

### Functional imaging results of the sequential finger-tapping task

The main effect of the sequential finger tapping showed increased BOLD signal in controls and patients on both sides of the cerebellum lobulus VI, temporal lobe, the middle and superior frontal cortex, supplementary motor area, sensorimotor cortex, right cingulum, inferior and superior parietal lobe, middle occipital lobe, the insula, putamen, and thalamus (Table 2 for details). There was no group difference on whole-brain analysis.

**Table 2:** Main effects of tapping in all participants. Peak locations functional magnetic resonance imaging (fMRI; BOLD activity), after correction for multiple comparisons (FEW) *P* > 0.05 in all listed regions

	Left hemisphere				Right hemisphere			
	MNI peak coordinate				MNI peak coordinate			
Region	*x*	*y*	*z*	*T*-value	*x*	*y*	*z*	*T*-value
Precuneus					12	−66	56	12.18
Precentral	−56	8	22	15.72	56	8	36	12.48
Frontal Mid	−38	38	22	7.74	24	8	48	9.02
Frontal Sup	−20	6	62	8.61	24	−10	62	15.4
Postcentral	−60	−18	24	14.2	40	−20	52	18.21
Cingulum_Mid					6	6	44	13.13
Parietal_Inf	−46	−30	38	15.21	40	−34	48	17.19
Parietal_Sup	−38	−48	60	14.29	32	−48	58	15.22
Temporal_Mid	−46	−58	2	9.68	44	−58	6	9.42
Temporal Sup	−52	−36	18	13.60	60	−28	18	11.20
Occipital Mid	−46	−66	2	11.51				
Rolandic_Oper					60	−18	16	15.01
SupraMarginal	−56	−22	34	13.73				
Supp Motor Area	−6	−4	62	15.12	6	−2	62	15.13
Frontal Inf Oper	−56	8	16	16.50	52	10	10	18.49
Temporal Pole Sup	−52	6	2	14.37				
Cerebellum Lobulus IV	−16	−54	−22	23.43	20	−56	−24	18.12
Vermis					4	−64	−16	16.32
Insula	−34	22	0	9.36	38	18	−2	11.73
Putamen	−22	2	0	9.16	26	0	8	11.79
Thalamus	−12	−16	2	9.27	14	−18	2	10.49

**Figure 3:**
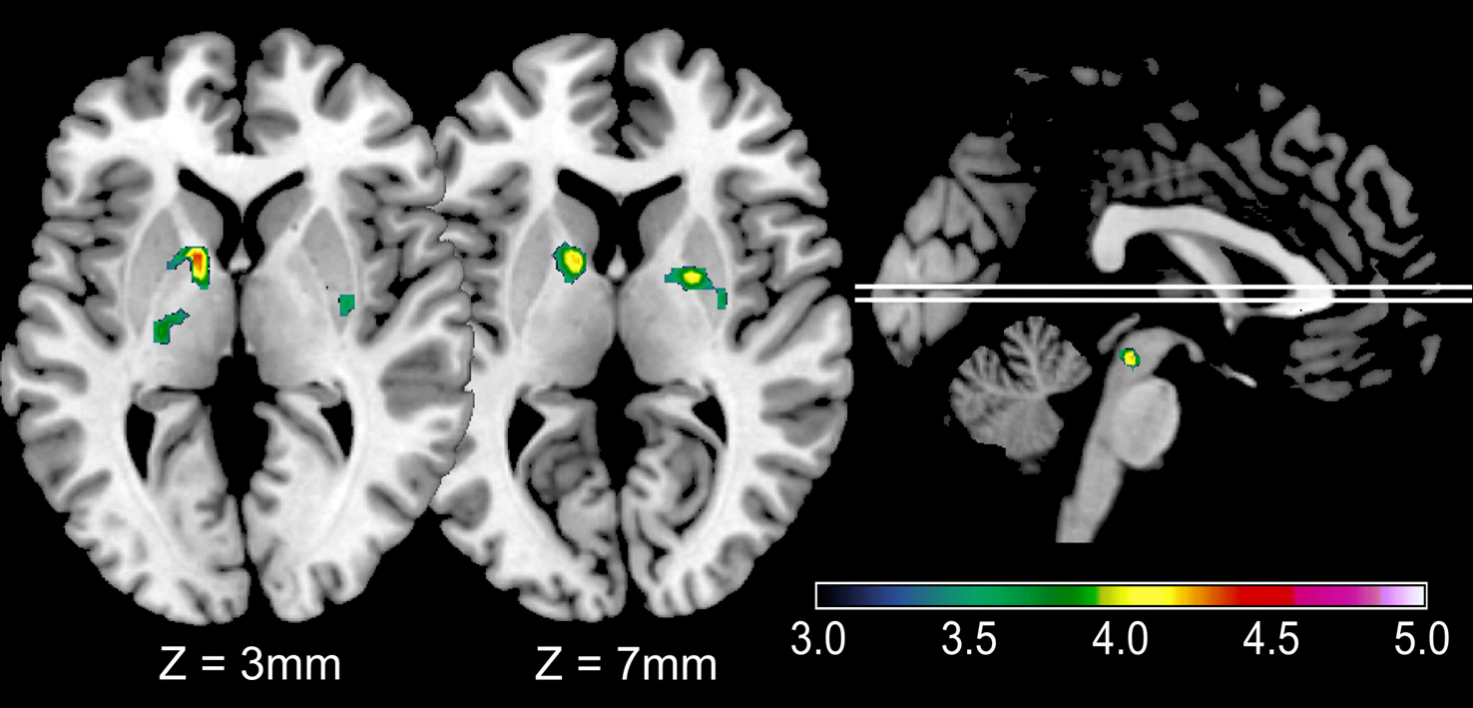
Statistical maps of the functional magnetic resonance imaging (fMRI) analysis from the sequential tapping task: Patients exhibited reduced blood oxygen level-dependent (BOLD) signal in the right anterior putamen (peak increase right: *x*,*y*,*z *=* *22, 0, 6; *ρ*SVC/FWE = 0.02) and left anterior globus pallidus (peak increase left: *x*,*y*,*z *=* *−12, 6, 4; *ρ*SVC/FWE = 0.01) compared to controls. The figure shows the results of the whole-brain analysis without any masking.

Differences in BOLD signal were detected between the two groups only when restricting the comparison to the putamen and the globus pallidus using the small volume correction. Patients exhibited reduced BOLD signal in the right anterior putamen/globus pallidus (peak difference right: *x*,*y*,*z *=* *22, 0, 6; *ρ*_SVC/FWE_ = 0.02) and the left anterior globus pallidus (peak difference left: *x*,*y*,*z *=* *−12, 6, 4; *ρ*_SVC/FWE_ = 0.01; see Table 3 and Fig.3). Disease duration showed no relation to the BOLD signal in the patient group. Both the tapping frequency (peak increase right sensorimotor cortex: *x*,*y*,*z *=* *38, −24, 54; *ρ*_SVC/FWE_ < 0.001) and the number of correct sequences (peak increase right sensorimotor cortex: *x*,*y*,*z *=* *38, −24, 56; *ρ*_SVC/FWE_ = 0.03) correlated positively with BOLD signal in the sensorimotor cortex, but there were no significant differences between the groups.

**Table 3:** Peak locations of clusters in statistical maps. Results of functional magnetic resonance imaging (fMRI)-tapping group (BOLD activity) and voxel-based morphometry (VBM) group and regression analysis. *P*-values represent effects after small volume correction (SVC), defined by the combined region of the putamen and globus pallidus extracted from the AAL atlas (Tzourio-Mazoyer et al. 2002)

	MNI peak coordinate						
Region	*x*	*y*	*z*	Cluster extend (voxel)	*Z*-score	*T*-value	p_FWE_ (SVC correction)
fMRI: BOLD activity decreased (writer’s cramp patients < healthy controls)							
Right Putamen/Pallidum	22	0	6	134	3.79	4.12	0.0231
Left Putamen/Pallidum	−12	6	4	96	4.01	4.40	0.0108
VBM: Dystonia-related increases in gray matter volume (writer's cramp patients > healthy controls)							
Right Putamen/Pallidum	26	−10	0	104	3.70	4.03	0.0297
Left Putamen/Pallidum	−22	−4	0	183	3.61	3.92	0.0401
VBM: Negative correlation between disease duration and gray matter volume in patients							
Right Putamen/Pallidum	24	4	6	110	3.97	5.21	0.0126
Left Putamen/Pallidum	−24	4	8	80	3.93	5.11	0.0147

We further analyzed differences in BOLD effect in the tapping condition at the beginning of the learning process (blocks 1–7) compared to the effect at the end of the experiment (blocks 8–15), and observed a decrease in BOLD signal in the visual cortex (calcarine sulcus), the precuneus and vermis in both groups (Fig. S1, supplemental material). A correlation analysis of the BOLD differences and the learning parameter *r* showed no differences between the two groups. Our analyses with only good learners showed similar results.

### Morphometric imaging results

#### Group differences

**Figure 4:**
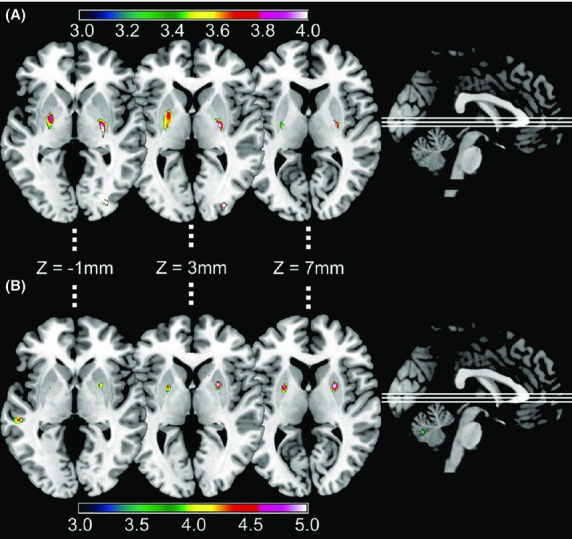
Visualization of the local gray matter density analysis: (A) Patients showed increased gray matter density bilateral in the dorsal putamen and globus pallidus, which was more pronounced on the right side (peak increase right: *x*,*y*,*z *=* *26, −10, 0; *ρ*SVC/FWE = 0.03; peak increase left: *x*,*y*,*z *=* *−22, −4, 0; *ρ*SVC/FWE = 0.04). (B) Patients with longer disease duration exhibited less increase of gray matter density in the putamen and globus (peak increase right: *x*,*y*,*z *=* *24, 4, 6; *ρ*SVC/FWE = 0.013; peak increase left: *x*,*y*,*z *=* *−24, 4, 8; *ρ*SVC/FWE = 0.015).

According to our hypothesis the VBM analysis of the basal ganglia there was an increase of gray matter volume in the posterior parts of the putamen and globus pallidus bilateral in patients with writer's cramp compared to controls. This increase of volume was more pronounced on the right side (peak increase right: *x*,*y*,*z *=* *26, −10, 0; *ρ*_SVC/FWE_ = 0.03; peak increase left: *x*,*y*,*z *=* *−22, −4, 0; *ρ*_SVC/FWE_ = 0.04; see Table 3 and Fig.4A).

Extended exploration of the whole brain with a correction for multiple comparisons revealed no further significant differences in local gray matter volume between the groups.

#### Regression model with disease duration

In the regression model with the disease duration, we found a negative correlation between the duration of symptoms and the local gray matter volume (peak increase right: *x*,*y*,*z *=* *24, 4, 6; *T *=* *5.21; *ρ*_SVC/FWE_ = 0.01; peak increase left: *x*,*y*,*z *=* *−24, 4, 8; *T *=* *5.11; *ρ*_SVC/FWE_ = 0.02; see Table 3, Fig.4B and supplemental material Fig. S2). In other words, in patients, the increased volumes seem to be reduced to a more normal level with disease progression. Additionally, there was a negative correlation between age and gray matter volume in the same region of interest (peak increase right: *x*,*y*,*z *=* *26, 16, 0; *T *=* *4.21; *ρ*_SVC/FWE_ < 0.06; peak increase left: *x*,*y*,*z *=* *−24, 6, 8; *T *=* *4.45; *ρ*_SVC/FWE_ < 0.04; Fig. S3 supplemental material). The two variables, age and disease duration, were not correlated (*P* > 0.5) in our study.

## Discussion

In this study, we combined functional and structural imaging analyses in patients with writer's cramp. With motor practice, both groups showed similar, significant improvement. As expected, sequential finger tapping induced bilateral activity in both groups in the cerebellum, the supplementary motor area, sensorimotor cortex, and the insula. After small volume correction writer's cramp patients demonstrated reduced activity in the right anterior putamen/globus pallidus and the left anterior globus pallidus. Clinical parameters and BOLD response exhibited no correlation. The BOLD signal in the sensorimotor cortex correlated positively with the tapping frequency and the number of correct responses without any group differences. Since we measured the BOLD signal during the learning phase (Doyon and Benali 2005; Dayan and Cohen 2011) of a complex sequential tapping task, we compared the first with the second half of the experiment in a further separate analysis (Grafton et al. 2002; Floyer-Lea and Matthews 2005; Sun et al. 2007). In both groups, the BOLD signal in the visual cortex decreased in the late learning phase. A possible explanation is that with practice motor performance automatizes and becomes less dependent from visual instructions during the experiment.

Gray matter volume differences showed higher contrasts in the posterior putamen and globus pallidus bilaterally after small volume correction. Patients with longer disease duration exhibited fewer differences in gray matter volume compared to those with shorter disease duration.

### Functional MRI

There are several different aspects that might account for the results in our study: First, the complexity of a task influences the extent and magnitude of brain activations and has to be respected in this study. Simple motor tasks induced activation of the contralateral sensorimotor cortex (Cramer et al. 1999) and the ipsilateral cerebellum (Catalan et al. 1998; Gerloff et al. 1998; Verstynen et al. 2005). In writer's cramp patients, a simple, repetitive left index flexion task was associated with less activation bilateral in the supplementary motor area (Islam et al. 2009). In contrast, complex sequential finger movements in healthy volunteers lead to an activation in the ipsilateral sensorimotor cortex, the supplementary motor area, premotor area, bilateral posterior parietal area, and the precuneus (Shibasaki et al. 1993; Catalan et al. 1998; Cramer et al. 1999; Verstynen et al. 2005). In patients with focal hand dystonia, a complex tapping task included either an alternating opposition of fingers II–V to the thumb or a sequential finger-tapping task and caused decreased cortical (Islam et al. 2009), thalamical, and basal ganglia activation (Kadota et al. 2010), specifically in the putamen (Wu et al. 2010). We clearly used a complex, sequential finger-tapping task and demonstrated diminished activity in the contralateral anterior putamen/ipsilateral globus pallidus, which is in agreement with these previous studies. Double-sided underactivation might be attributed to the complexity of the task, because such tasks tend to use widely distributed networks involving both hemispheres (Hausmann et al. 2004). On the other hand, our findings underline the bilateral functional impairments in the basal ganglia in writer's cramp. Previous studies demonstrated bilateral activation of the cortex and the striatum after sequential left-hand finger tapping (Mattay et al. 1998), and the putamen seems to be particularly involved in nonroutine left-handed movements (Francois-Brosseau et al. 2009). However, it is unlikely that tapping with the left hand can solely be attributed to our findings, because the paradigm was equal in patient and controls and cannot account for the underactivation in the putamen/globus pallidus only in our patient group.

Second, we have to account for the effect of learning in our study. Previously, training that lasted for more than 10 min was considered long-term training. It produced decreased BOLD signal in the anterior, associative putamen accompanied by an increase in the posterior, sensorimotor putamen (Lehericy et al. 2005). In our study, the training lasted 15 min and corresponded more to that longer than the early learning phase. In an additional analysis, we separated an early (blocks 1–7) from a late learning phase (blocks 8–15) to compare the two phases and discovered no abnormalities in the basal ganglia or cerebellum of patients. Thus, the underactivation in BOLD signal in the anterior part of the putamen/globus pallidus in our study can probably not be attributed to a difference of learning effect in our study. However, a limitation of our study is that the control task to evaluate the specific effect of learning as it had been included by Lehericy et al. (Lehericy et al. 2005), has not been performed.

Third, the anterior (associative) part of the putamen is not only associated with the learning phase, but also with nonroutine planning. Nonroutine novel movement sequences caused activation in the caudate nuclei (heads) and adjacent parts of the anterior putamen bilaterally (Jankowski et al. 2009). In this study, we applied a left-handed, nonroutine movement sequence and detected abnormally decreased activity in the contralateral, anterior putamen/globus pallidus in patients. Hence, sensorimotor planning seems to be disturbed, even when using the healthy hand, a phenomenon that supports the view of a primary bilateral disturbance. The ipsilateral BOLD signal reduction in the globus pallidus might be attributed to the particular importance of the left hemisphere during the planning phase of complex movements (Ziemann and Hallett 2001; Serrien et al. 2006; Bohlhalter et al. 2009; Wheaton et al. 2009; van den Berg et al. 2011).

Recently, the connection between anterior and posterior putamen to cortical areas in patients with task-specific dystonia was determined. Connectivity was increased from the anterior putamen to nonprimary motor and cerebellar areas that are involved in motor planning (Moore et al. 2012). One concept is a deficiency of GABAergic interneurons in the striatum that could be responsible for decreased motor surround inhibition, a process that is important for selective motor tasks (Hallett 2011). In our study, patients showed no disturbed individuated finger control in the tapping task using their left hand; therefore the results cannot be explained by a deficit in motor performance. The underactivation of the anterior putamen and globus pallidus could reflect a dysfunctional output in the indirect pathway that may lead to an overactivity of the excitatory direct pathway. The result is an increased output to the thalamus and motor cortex, which may be a contributor to impaired surround inhibition. This concept had been put forward recently (Hallett 2011). An alternative possible assumption is that the underactivation of the anterior putamen could also be regarded as a compensatory mechanism for increased excitability in areas attributed to motor planning. However, these conclusions remain speculative.

A fourth aspect to integrate our findings into current concepts is the relevance of dopaminergic mechanisms for movement planning (Reeves et al. 2005). Previous studies confirmed striatal dopamine receptor deficiency in dystonia (Perlmutter et al. 1997; Tanabe et al. 2009). In writer's cramp D_2_/D_3_ availability was reduced at rest, and during a right finger-tapping task-deficient dopamine release displayed in the bilateral putamen and left nucleus caudatus (Berman et al. 2013). Therefore, the reduction of BOLD activity in the right anterior putamen and the left anterior globus pallidus in writer's cramp patients in our study could also result from dopamine receptor deficiency.

Finally, the cerebellum, which is crucial for selection, preparation, and execution of complex motor tasks, influences basal ganglia activity and is dysfunctional in dystonia (Neychev et al. 2008; Kadota et al. 2010; Wu et al. 2010; Fan et al. 2012). Cerebellar mechanisms are involved in adjusting movement kinematics according to sensory input to produce accurate motor output during early learning (Penhune and Doyon 2002). Reduced activation in the left cerebellum has been described in patients with musician's (Kadota et al. 2010) and writer's cramp during simple and complex tapping tasks (Wu et al. 2010). In contrast to our expectations, our study indicated no differences in the cerebellum, regardless of the learning performance or phase. This discrepancy is not entirely clear, but possibly tapping with the nonaffected hand may uncover a generalized pathophysiology, while compensatory mechanisms may not (yet) occur.

### Structural MRI

In contrast to the fMRI results, voxel-based morphometry showed increased gray matter volume bilateral in adjacent areas, but no clear correlation. Previous studies also described increased gray matter in the putamen and globus pallidus in patients with focal dystonia (Black et al. 1998; Draganski et al. 2003), and in musician's cramp (Granert et al. 2011). In contrast, decreased gray matter density was found in the left primary sensorimotor cortex, the cerebellum bilaterally, and the thalamus in 30 patients with writer's cramp (Delmaire et al. 2007). However, the total gray matter volume in that study was significantly greater in the group of patients. The authors attribute methodological factors such as differences in the populations studied or the method used for data analysis for these discrepancies.

The activities of the dorsal and medial regions of the globus pallidus are influenced by cognitive components during motor tasks. These areas receive input from the dorsal putamen and the caudate and project to prefrontal and premotor areas of cortex, and the supplementary motor area (Mushiake and Strick 1995). Dystonic patients show increased connectivity from the posterior part of the globus pallidus to motor and premotor regions (Rozanski et al. 2013). It is conceivable that as a result of impaired movement automatization, writer's cramp patients need more cognitive elements to perform complex motor tasks. This could explain the increase of gray matter in the basal ganglia as a secondary phenomenon in our study. Further possible explanations include muscle cocontraction and overflow as well as cortical overactivity with increased cortical input. However, a primary phenomenon cannot be excluded.

Patients with longer disease duration demonstrated less gray matter density in the putamen and globus pallidus, which could reflect a compensation of the basal ganglia circuit after long time of malfunction in this system.

In summary, patients with writer's cramp showed a decreased BOLD response in the right anterior putamen and the left globus pallidus during a complex motor task of the healthy hand after applying small volume correction restricted to the putamen and the globus pallidus. The structural analysis showed increased gray matter density in adjacent areas bilateral, but without a clear correlation between the functional and structural findings. With the methods we used we cannot distinguish, whether our results have to be attributed to an adaptation or compensation of the basal ganglia circuit after many years of dystonic cocontraction or reflects a primary problem that could be genetic or even an endophenotypic trait.

## Supporting Information

Figure S1 Statistical maps of the fMRI analysis from the sequential tapping task: Regions with decreased BOLD effect in the tapping condition from the beginning of the learning process (blocks 1–7) to the end of the experiment (blocks 8–15). For visualization, a statistical threshold of punc < 0.001 is used. Outlines: red = precuneus, green = vermis, blue = calcarine, purple = lingual.

Figure S2 Regression analysis between disease duration and the local gray matter volume estimates of the left (blue) and right (red) putamen. Gray matter estimates were extracted at the peak locations of the statistical map. Shorter disease duration correlated with higher gray matter volume in the putamen with this single factor explaining 50% (right) and 23% (left) of the variance.

Figure S3 Regression analysis between age and the local gray matter volume estimates of the left (blue) and right (red) putamen. Gray matter estimates were extracted at the peak locations of the statistical map. Younger age correlated with higher gray matter volume in the putamen with this single factor explaining 7% (right) and 19% (left) of the variance.
