# Supplementary figures and images for: Increased volume and impaired function: the role of the basal ganglia in writer’s cramp
Source: Brain Behav. 2014 Dec 24;5(2):e00301. doi: 10.1002/brb3.301 (PMC4309880; doi:10.1002/brb3.301)

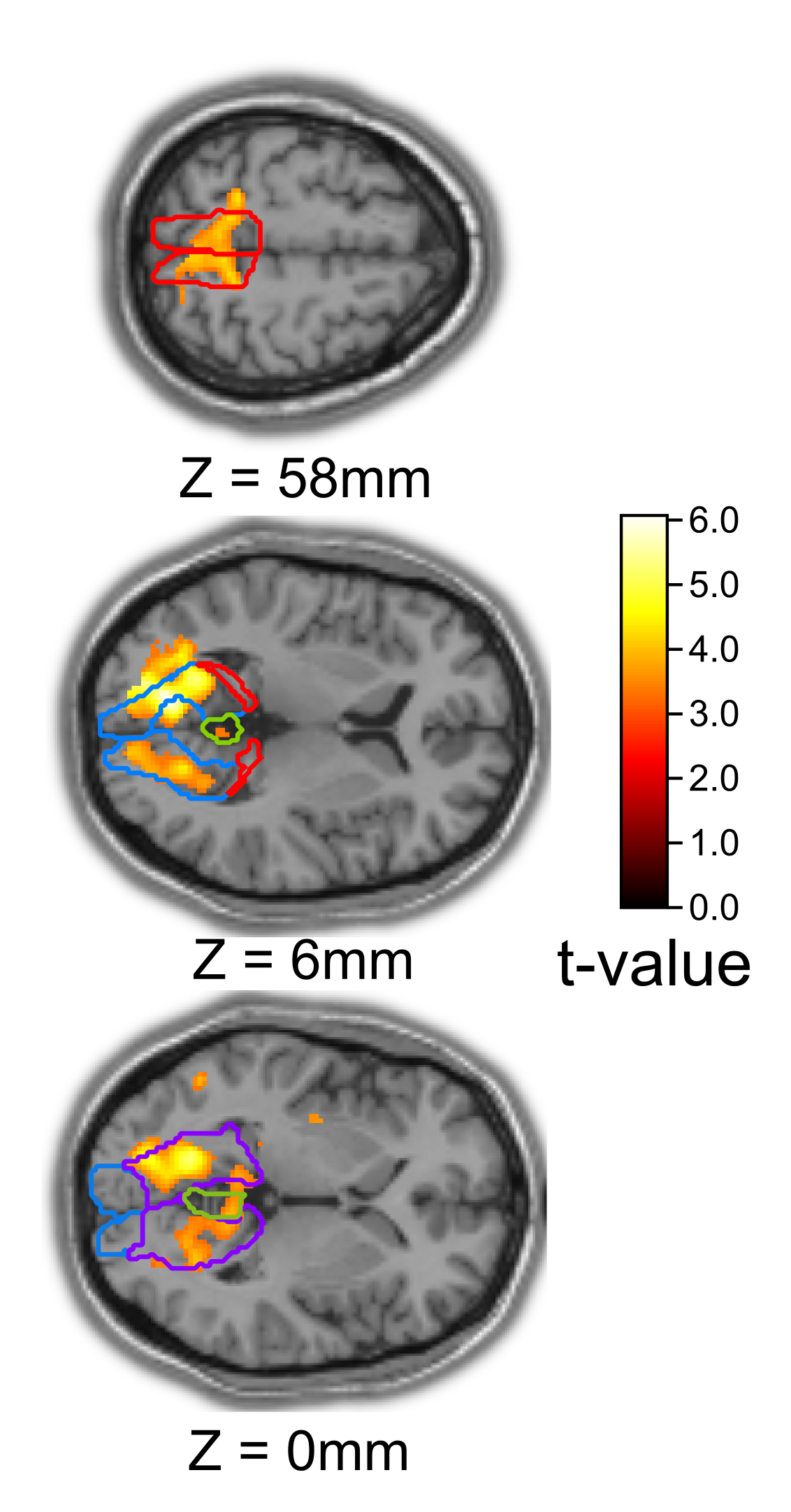

Supplement: Figure S1 — Statistical maps of the fMRI analysis from the sequential tapping task: Regions with decreased BOLD effect in the tapping condition from the beginning of the learning process (blocks 1–7) to the end of the experiment (blocks 8–15). For visualization, a statistical threshold of punc < 0.001 is used. Outlines: red = precuneus, green = vermis, blue = calcarine, purple = lingual. [file brb30005-e00301-sd1.tif]

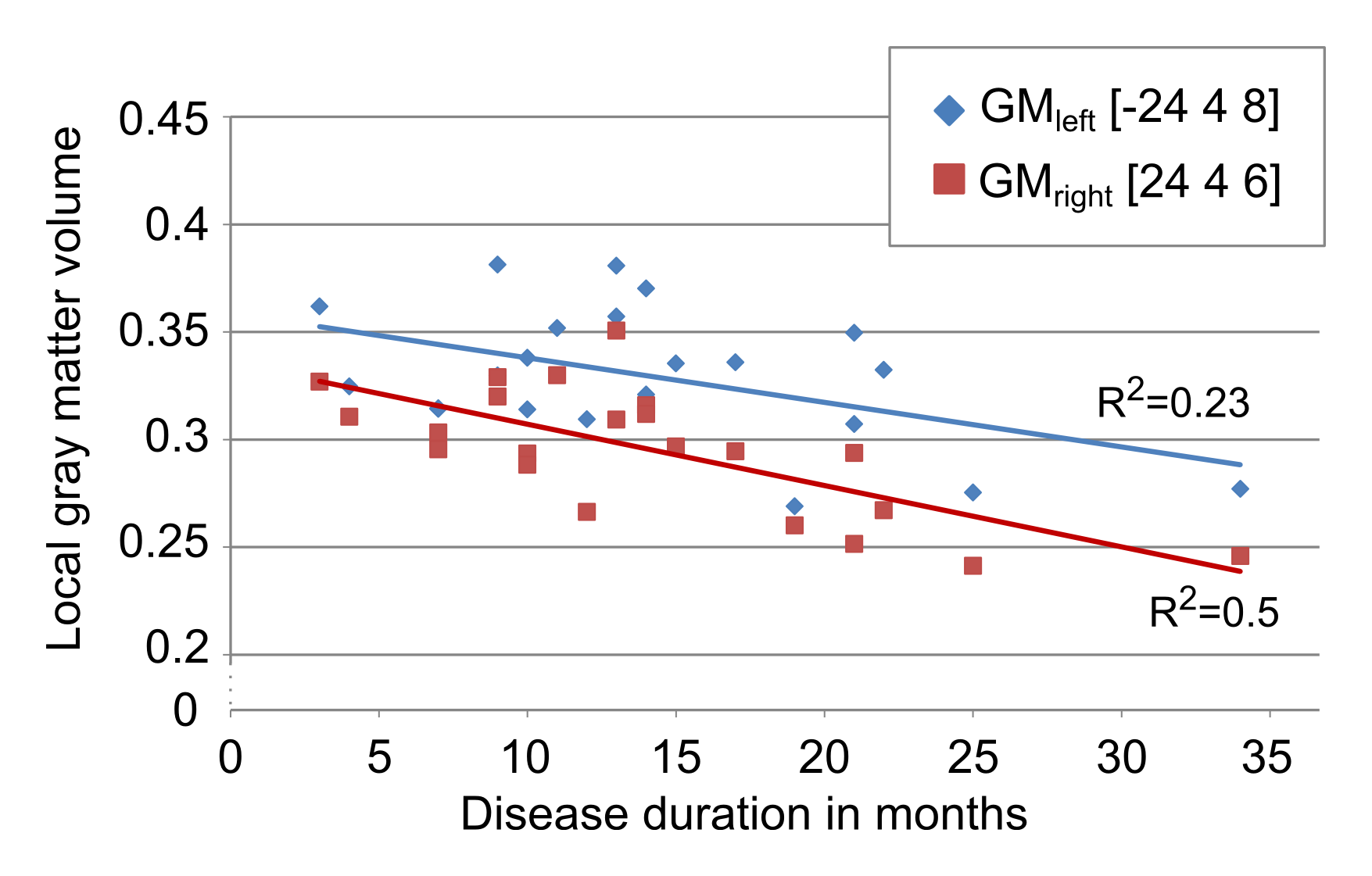

Supplement: Figure S2 — Regression analysis between disease duration and the local gray matter volume estimates of the left (blue) and right (red) putamen. Gray matter estimates were extracted at the peak locations of the statistical map. Shorter disease duration correlated with higher gray matter volume in the putamen with this single factor explaining 50% (right) and 23% (left) of the variance. [file brb30005-e00301-sd2.tif]

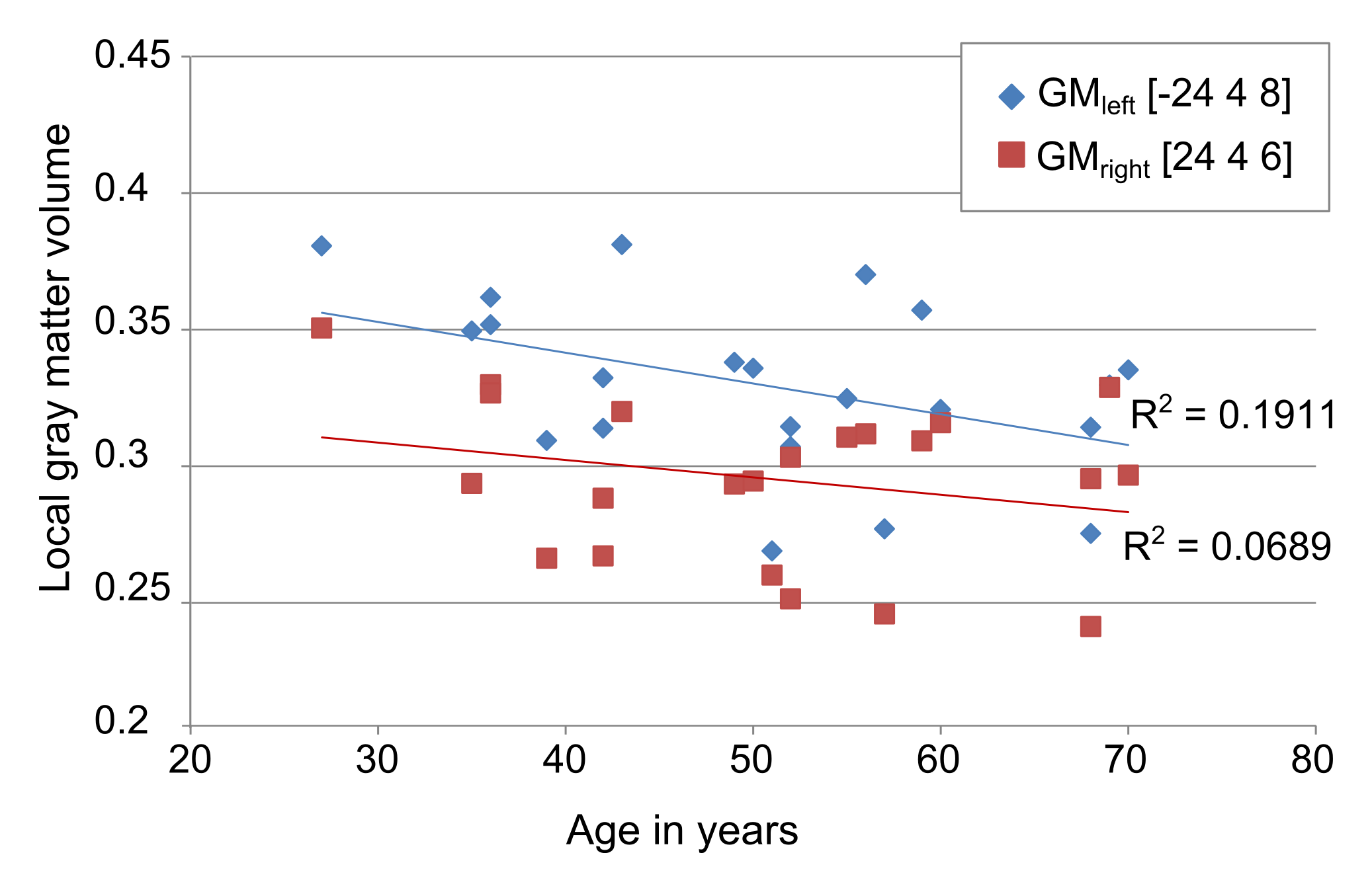

Supplement: Figure S3 — Regression analysis between age and the local gray matter volume estimates of the left (blue) and right (red) putamen. Gray matter estimates were extracted at the peak locations of the statistical map. Younger age correlated with higher gray matter volume in the putamen with this single factor explaining 7% (right) and 19% (left) of the variance. [file brb30005-e00301-sd3.tif]
